# Does arts and cultural group participation influence subsequent well-being? A longitudinal cross-country comparison of older adults in Japan and England

**DOI:** 10.1136/bmjph-2023-000865

**Published:** 2024-08-24

**Authors:** Jessica K Bone, Taiji Noguchi, Hei Wan Mak, Daisy Fancourt, Katsunori Kondo, Tami Saito

**Affiliations:** 1 Department of Social Science, Center for Gerontology and Social Science, Research Institute, National Center for Geriatrics and Gerontology, Obu, Aichi, Japan; 2 Japan Society for the Promotion of Science, Chiyoda-ku, Japan; 3 Behavioural Science and Health, Institute of Epidemiology and Health Care, University College London, London, UK; 4 Department of Community Building for Well-being, Center for Preventive Medical Sciences, Chiba University, Chiba, Chiba, Japan; 5 Research Department, Institute for Health Economics and Policy, Tokyo, Japan

**Keywords:** Public Health, Community Health, Social Medicine

## Abstract

**Introduction:**

Arts engagement is a positive health behaviour that could support the mental and social well-being of ageing populations globally. However, research is predominantly from Western countries, leaving it unclear whether arts engagement can support well-being in Japan, where arts are differently valued and engaged with. The social gradient in arts engagement and well-being may also have led to an overestimation of the impact of participation on well-being in Western countries. We therefore tested whether participation in community arts and cultural groups was associated with subjective well-being and social support after removing confounding by demographic, socioeconomic and health-related factors in Japan and England.

**Methods:**

We harmonised longitudinal data from the Japan Gerontological Evaluation Study (JAGES) 2016 and 2019 waves and the English Longitudinal Study of Ageing (ELSA) 2014 and 2018 waves to enable cross-country comparisons. We included 9511 adults aged ≥65 years from JAGES and 3133 participants aged ≥65 years from ELSA. Using inverse probability-weighted regression adjustment, we estimated the effect of arts and cultural groups on subsequent life satisfaction, happiness and depressive symptoms (subjective well-being) as well as social support.

**Results:**

In JAGES, arts and cultural group participation was associated with higher odds of life satisfaction and higher social support scores. In ELSA participants aged ≥65 years, group participation was only associated with higher depressive symptoms. But, in a sensitivity analysis with the full ELSA sample aged ≥50 years (n=5128), this association was no longer present. Instead, group participation was associated with higher social support scores.

**Conclusion:**

Our findings indicate that arts and cultural group participation can enhance life satisfaction and social support in Japan, with small but more consistent benefits than in England. Facilitating participation in arts and cultural groups could help older adults to maintain a healthy social support network, which may further support their health as they age.

**WHAT IS ALREADY KNOWN ON THIS TOPIC**

**WHAT THIS STUDY ADDS**

- We provide the first evidence that, independent of a range of demographic, socioeconomic, neighbourhood and health-related factors, arts and cultural group participation can enhance life satisfaction and social support in Japan, with small but perhaps more consistent benefits than in England. Despite differences in artistic expression and funding, arts and cultural groups may enable older adults across countries to maintain a healthy social support network.

**HOW THIS STUDY MIGHT AFFECT RESEARCH, PRACTICE OR POLICY**

- Our mixed evidence indicates the need for further research with more detailed multidimensional measures of well-being (particularly in England). Despite this, findings provide some support for increasing access to arts and cultural groups for older adults in Japan, such as through community salons or by implementing social prescribing.

## Introduction

 Globally, the number of older adults is increasing more rapidly than any other age group. Japan has one of the largest ageing populations in the world, with more than one in four people aged 65 years and over.^1^ The ageing population is at increased risk of physical and mental health problems, dementia and social isolation.^2^ Consequently, older adults may experience decreases in subjective well-being (how they experience and evaluate their lives),^3^ as well as social well-being (the quality of relationships with other people).^4^ Identifying accessible and cost-effective strategies to support older adults’ well-being is thus a public health priority.^5^

Arts and cultural groups (such as music, educational and crafts groups) may enhance older adults’ well-being as they involve a range of health-promoting activities, including opportunities for social interactions, creative expression, cognitive stimulation, physical activity, collaborative learning and developing self-esteem.^6^,^8^ Since 2015, the Japanese government has focused on increasing community-based support networks for older adults through the development of community salons.^9^,^12^ Community salons offer regular group activities for older adults, including arts and cultural activities. Although there is some evidence that engagement in community, arts and cultural activities can reduce depressive symptoms in older adults in Japan,^13^,^15^ this research has not been extended to other aspects of well-being.

In Western countries, systematic and scoping reviews have identified broad evidence for associations between arts and cultural engagement, subjective well-being, social support and quality of life in older adults.^816^,^18^ Population-based studies of older adults have also generally found benefits for well-being, although evidence is not always consistent.^19^,^22^ However, little is known about whether these associations are present outside of Western countries. The contrasting socio-cultural structure and context in Japan means the arts are differently valued, which may alter their impact on well-being. Artistic value is constructed through societal attitudes and traditions, and there are substantial variations in artistic expression, aesthetic experiences, arts administration and funding across Western countries and Japan.^23^,^25^ In England, much funding for the arts is administered by the Arts Councils, which are ‘arm’s length bodies’ operating independently from the government. Central government and local authorities have a greater role in Japan, particularly in approving cultural value, with an emphasis on traditional art forms through the designation of objects and heritage as ‘National Treasures’. There is also less support for community or participative arts in Japan.^23^

Additionally, across Western countries and Japan, people with higher education and socioeconomic position are more likely to engage in arts.^26^,^28^ As there are similar socioeconomic disparities in well-being,^29^ previous research in Western countries may have overestimated the impact of arts engagement on well-being due to confounding. Although studies have generally adjusted regression models for various demographic and socioeconomic factors, this can leave residual imbalances between those who do and do not engage in arts and bias results.^30^ Socioeconomic position is a complex construct that is difficult to measure. Some studies have employed more sophisticated methods to address confounding, such as fixed effects regression and propensity score matching, but most have been in younger adults.^1931^,^33^ Reverse causality may also have biased estimates, as enhanced well-being leads to higher subsequent social engagement, including in arts and cultural activities.^34^ It therefore remains a priority to determine whether any beneficial effects of arts engagement for older adults are independent of the protective effects of broader structural, functional and social factors.

In this study, we tested whether arts and cultural groups can support the well-being of older adults in Japan and England. We had two overarching aims: (i) to estimate the effect of arts and cultural groups on subsequent subjective well-being and social support in Japan and (ii) to explore whether findings from Japan are similar to those from Western countries such as England. We specifically focused on Japan and England as high-income countries with ageing populations, where identifying strategies to support older adults’ well-being is a priority. These countries provide contrasting contexts for testing the effects of arts and cultural groups, with differences in culture, artistic value and funding for the arts. If arts and cultural groups consistently support well-being in these diverse contexts, this would provide more robust evidence for a causal relationship. Measures of arts and cultural group participation and well-being were also well aligned across studies in these countries, facilitating this comparison. By directly comparing findings using harmonised data from Japan and England, we aimed to indicate whether evidence from Western countries is applicable to East Asian countries. We operationalised subjective well-being using measures of life satisfaction, happiness and depressive symptoms and measured perceived social support. We used doubly robust estimators to remove confounding by demographic, socioeconomic and health-related factors. We hypothesised that engaging in arts and cultural groups would be associated with enhanced subjective well-being and social support in Japan and England.

## Methods

### Sample

#### Japan

The Japan Gerontological Evaluation Study (JAGES) is a large-scale population-based longitudinal study established in 2010, with data mainly collected through self-administered mail surveys. It includes people aged 65 and older who do not receive long-term care insurance benefits in Japan.^35^ JAGES has conducted surveys every 3–4 years in municipalities that are the public providers of long-term care insurance. Although municipalities were not selected randomly, JAGES has been conducted nationwide from very rural to metropolitan municipalities, with the sample from each municipal unit selected randomly or by complete enumeration. We used data from Wave 3 (2016), our baseline, and Wave 4 (2019), our follow-up. These waves were chosen as they included the most recent data available at the time of analysis and were the only waves to include similar measures of arts and cultural group participation to those used in England. Of the 180 005 participants with valid responses in Wave 3, 77 028 also participated in Wave 4 (not all participants from Wave 3 were eligible for Wave 4). We included only participants from the random subsample eligible to complete questions on arts engagement (n=9600; 12% of the total sample). We then further limited the sample to participants with no missing data on population weighting variables (age, sex, marital status, household size; n=89 excluded), leaving a final analytical sample of 9511 participants.

#### United Kingdom

The English Longitudinal Study of Ageing (ELSA) started in 2002/03 and follows over 11 000 participants aged 50 years and above every 2 years. It includes a nationally representative sample of older adults living in private households in England. We used data from Wave 7 (2014), our baseline, and Wave 9 (2018), our follow-up. These waves were chosen as they included the most recent data available and were most comparable to JAGES in terms of follow-up period and measures used. Participants were eligible for inclusion if they returned the ELSA self-completion survey in both Wave 7 and Wave 9, which included questions on arts engagement and well-being. Of the 8197 participants in the Wave 7 self-completion subsample, 5887 were also in the Wave 9 self-completion subsample. We retained only participants with valid self-completion weights at Wave 7, indicating their eligibility for this survey (n=5128). We further limited this sample to participants aged 65 and over, in line with JAGES, leaving a final analytical sample of 3133 participants.

### Patient and public involvement

It was not possible to include patients or the public in this secondary data analysis.

### Measures

#### Arts and cultural group participation

We created a binary indicator of arts and cultural group participation (yes, no) at baseline in both samples. In JAGES, this included participating in a study, learning or cultural group at least once a month or participation in any handicrafts, musical activities or writing activities at a community salon. In ELSA, group participation was indicated by membership of education, arts or music groups or evening classes. Further details on the measurement and harmonisation of all variables are included in online supplemental table 1.

#### Well-being

We used three measures of subjective well-being at baseline and follow-up in both samples: life satisfaction, happiness and depressive symptoms. We selected these outcomes as they were the only measures of well-being available in JAGES and were harmonisable with ELSA, and life satisfaction and happiness are two of the four items used to monitor population-level subjective well-being in the UK.^36^ Measuring depressive symptoms captures experienced well-being in terms of negative affect and psychological distress.^21 37^

A binary indicator of life satisfaction was created using one item. In JAGES, participants were asked whether they were satisfied with their current life (yes, no). In ELSA, participants were asked how satisfied they were with their lives nowadays, rated on an 11-point scale from not at all (0) to very (10). Responses were grouped to indicate being satisfied with life (ratings 5–10) versus not satisfied (ratings 0–4). This cut-off has previously been used in England to indicate low life satisfaction.^38^

Happiness was measured continuously with one item. In JAGES, participants rated the degree to which they were currently happy from 0 (very unhappy) to 10 (very happy). In ELSA, participants rated how happy they felt yesterday, from 0 (not at all) to 10 (very). Happiness was standardised within each analytical sample (mean=0, SD=1). A standardised score represents the number of SD each participant’s raw score is from the overall mean of that measure.

Depressive symptoms were measured continuously on validated scales. In JAGES, the 15-item Geriatric Depression Scale (GDS) measures depressive symptoms, with total scores ranging from 0 to 15.^39^ In ELSA, the eight-item Centre for Epidemiologic Studies Depression Scale (CES-D) was used, with total scores ranging from 0 to 8.^40^ Depressive symptoms were standardised in both samples. Higher scores indicate more severe depressive symptoms.

Perceived social support was measured using a harmonised index. In both samples, scores were summed on five binary items (total score 0 to 5). These items measured whether participants met up with friends at least once a month and could open up to their spouse, children, other family and friends about worries or concerns. Social support was standardised, with higher scores indicating more social support.

#### Covariates

We included a range of demographic, socioeconomic, neighbourhood and health-related factors, measured at baseline and harmonised across JAGES and ELSA. See online supplemental table 1 for further details on the measurement and harmonisation of all covariates. Demographic factors were age (years and quadratic), sex (male, female), marital status (married, widowed, other) and household size (continuous number of people living in household). Ethnicity (White, other) was only measured in ELSA. Socioeconomic factors were education (≤9, 10–12, ≥13 years), employment status (employed, unemployed, retired), equivalized household income (continuous), total assets (four categories), home ownership (yes, no), subjective financial status (difficult, average, comfortable) and childhood socioeconomic position (SEP; lower, higher).

Neighbourhood factors included social cohesion (standardised score), area physical disorder (standardised score) and urbanicity (urban, suburban, rural). In JAGES, the area physical disorder was not measured in 2016 but was self-reported in the prior wave (2013). We therefore averaged these scores within the smallest region available (elementary school district) and used these area-level averages as indicators of area physical disorder in 2016. For areas not divided into elementary school districts, we used the comprehensive ward level.

Health-related factors were general health rating (excellent, good, fair/poor), number of long-term health conditions (0–8 from: high blood pressure, diabetes, cancer, lung disease, heart disease, stroke, dementia/Alzheimer’s disease, Parkinson’s disease), ever had psychiatric problems (yes, no; ELSA only), depression diagnosis (yes, no), number of difficulties with activities of daily living (0–2 from: walking 100 yards, washing or dressing) and number of difficulties with instrumental activities of daily living (0–4 from: preparing hot meals, shopping for groceries, making telephone calls, managing money).

### Statistical analyses

We first examined the sample characteristics and prevalence of group participation in both datasets. We then investigated whether participating in arts and cultural groups led to better subsequent well-being. To address the issue that certain types of individuals may be more likely to participate in arts and cultural groups, we used doubly robust estimators. Our models compared the well-being of individuals who participated in arts and cultural groups (‘treatment’) with those who did not participate (‘controls’).

We used inverse probability-weighted regression adjustment (IPWRA). This involved creating weights that were the inverse probabilities of treatment, estimated using all identified covariates and the baseline measure of each outcome. The outcome model was then estimated using these weights and adjusted for all covariates and the baseline measure of each outcome. This approach combines the strengths of creating a model for the treatment (meaning group participation no longer depends on covariates) with a model for the outcome (meaning the contribution of covariates to well-being is also accounted for), making it less susceptible to bias.^41^ Estimates are consistent when either the outcome or the treatment model is correctly specified, providing two chances to make valid inferences (instead of just one), as long as either the treatment group or outcome can be determined as a function of the covariates and there are no unobserved confounders.

Using IPWRA, we estimated the average treatment effect (ATE) in the population, which is the average difference in well-being if everyone in the population participated in arts groups versus no-one in the population participated in arts groups. Separate models were estimated for each of the four well-being outcomes in both samples using the Stata 17 *teffects* commands.^42^ The treatment was estimated with a logit model, binary outcomes (life satisfaction) with a logit model and continuous outcomes (happiness, depressive symptoms, social support) with a linear model. Cluster-robust standard errors were applied, with clustering by elementary school district (JAGES) or household (ELSA). Different area sizes were used in clustering due to data availability. Multiple household members were included only in some JAGES regions, but this information was not available, so the smallest area unit was applied in both studies. Given the large number of clusters in both datasets (2445 in ELSA, 845 in JAGES), results should be unbiased and comparable.^43^ Although ethnicity was measured in ELSA, it could not be included in analyses due to the small proportion of participants who were not White (3%).

For all analyses, we applied weights to make the samples representative. Probability weights were not available for JAGES. We therefore weighted the final analytical sample to match the characteristics of the Japanese population aged 65 and over according to age, sex, marital status, and number of household members (obtained from the population census)^44^ using the Stata *ebalance* package.^45^ To remove extreme variation, weights were trimmed to a maximum of the median plus six times the IQR and then adjusted so that the total summed to the number of participants.^46 47^ In ELSA, we used probability weights for the self-completion questionnaire provided in the data, which account for complex sampling strategies and non-response.

For participants with missing data, we imputed data using multiple imputation by chained equations.^48^ We used predictive mean matching and logistic, ordered logistic, multinomial logistic, and truncated linear regression according to variable types, generating 40 imputed datasets for each sample (maximum missing data 39%; online supplemental table 2). The imputation models included all variables used in analyses, probability weights, and cluster variables. Data were imputed separately by sex as we hypothesised that the association between group participation and well-being would differ according to sex.

#### Sensitivity analyses

In sensitivity analyses, we computed E-values as indicators of how robust the main findings were to potential unmeasured confounding,^49^ using the Stata *evalue* package.^50^ E-values indicate the degree of unmeasured confounding, conditional on the measured covariates, required to shift the observed associations to the null.

Additionally, we explored whether the effect of group participation on well-being differed according to sex. To do this, we repeated the main analyses, with effects estimated separately for males and females. In ELSA, due to the limited sample size of patients aged 65 and above, the analyses stratified by sex were performed in all participants aged 50 and over. Finally, we tested whether the main findings were replicated in the complete ELSA sample, including all participants aged 50 and over (n=5128).

## Results

After weighting the JAGES sample (n=9511), participants were on average 76.05 years old (SD=6.15), 57% were female, 63% married, 66% retired, 83% owned their home and 83% reported good or excellent health (table 1). Overall, 16% of participants engaged in an arts or cultural group. At baseline, 87% were satisfied with their lives (table 2), with a mean happiness rating of 7.27 (SD=1.92), a depressive symptom score of 2.84 (SD=3.01) and social support index score of 2.51 (SD=1.18).

**Table 1:** Descriptive statistics for both samples after imputation and weighting

	JAGESn=9511	ELSAn=3133
	**Mean (SD**)	
Age	76.05 (6.15)	72.91 (6.34)
Household size	2.41 (1.01)	1.88 (0.72)
Household income	¥20.56 (¥14.91)	£19.88 (£14.14)
Neighbourhood social cohesion	3.75 (0.66)	5.59 (1.23)
Neighbourhood deprivation	2.37 (0.14)	2.64 (1.23)
Long-term health conditions	0.79 (0.80)	1.11 (0.97)
Difficulties with ADLs	0.12 (0.36)	0.22 (0.53)
Difficulties with IADLs	0.10 (0.37)	0.12 (0.43)
	**Proportion**	
Female	57%	54%
Marital status		
Married	63%	68%
Widowed	24%	17%
Other	14%	15%
Education		
≤9 years	31%	10%
10–12 years	41%	60%
≥13 years	28%	30%
Employment status		
Employed	24%	13%
Unemployed	10%	5%
Retired	66%	82%
Total assets		
1	28%	17%
2	17%	29%
3	41%	31%
4	14%	22%
Home owner	83%	84%
Subjective financial status		
Difficult	28%	2%
Average	56%	19%
Comfortable	16%	79%
Higher childhood SES	60%	96%
General health		
Excellent	15%	9%
Good	72%	67%
Fair/poor	13%	24%
Psychiatric diagnosis	–	9%
Self-reported depression diagnosis	1%	5%
Participated in arts group	16%	14%

Note. Results weighted with cluster robust standard errors and based on 40 imputed datasets. Raw neighborhoodneighbourhood cohesion ranged from 1 to 5 in JAGES and 1 to 7 in ELSA. Raw neighborhoodneighbourhood deprivation ranged from 1 to 4 in JAGES and 1 to 7 in ELSA. Household income is in units of 100 000 yen in JAGES and £10 000 in ELSA. Total asset categories correspond to <5, 5–9.99, 10–49.99, and ≥50 million yen in JAGES and <100, 100–249, 250–499, and ≥500 thousand pounds in ELSA.

ADLactivities of daily livingELSAEnglish Longitudinal Study of AgeingJAGESJapan Gerontological Evaluation Study SESsocioeconomic status

**Table 2:** Well-being at baseline and follow-up

	JAGES (n=9511)		ELSA (n=3133)	
	Baseline	Follow-up	Baseline	Follow-up
	N (proportion)			
Life satisfaction	8273 (87%)	7916 (83%)	2849 (91%)	2841 (91%)
	Mean (SD)			
Happiness	7.27 (1.92)	7.19 (1.93)	7.62 (2.13)	7.67 (2.02)
Depressive symptoms	2.84 (3.01)	3.24 (3.17)	1.21 (1.67)	1.37 (1.72)
Social support	2.51 (1.18)	2.42 (1.15)	4.09 (0.90)	3.98 (0.97)

Note. Results weighted with cluster robust standard errors and based on 40 imputed datasets. Social support ranges from 1 to 5 in JAGES and 1 to 7 in ELSA. Depressive symptoms range from 0 to 15 in JAGES and 0 to 8 in ELSA.

In ELSA after weighting (n=3133), participants were on average 72.91 years old (SD=6.34), 54% were female, 68% married, 82% retired, 84% owned their home and 76% reported good or excellent health (table 1). Overall, 14% of participants engaged in an arts or cultural group. At baseline, 91% were satisfied with their lives (table 2), with a mean happiness rating of 7.62 (SD=2.13), a depressive symptom score of 1.21 (SD=1.72) and a social support index score of 4.09 (SD=0.90).

### Subjective well-being

In JAGES, arts and cultural group participation was associated with 1.04 times higher odds of being satisfied with life compared to not participating (ATE OR)=1.04, 95% CI (CI)=1.004, 1.08). This OR translated to 86.4% of the treatment group being satisfied with life, relative to 86.0% of the control group at baseline. There was also very weak evidence that group participation was associated with a 0.08 SD lower depressive symptom score versus not participating (ATE coef=−0.08, 95% CI=-0.16, 0.005). There was no evidence for an association between group participation and subsequent happiness (table 3).

**Table 3:** Results from inverse probability-weighted regression adjustment estimating the effect of group arts engagement on subsequent well-being

	JAGES (n=9511)		ELSA (n=3133)	
	ATE (95% CI)	p value	ATE (95% CI)	p value
	Binary outcome: OR			
Life satisfaction	**1.04(1.004, 1.08**)	**0.031**	0.99 (0.94, 1.03)	0.544
	Continuous outcome: Coef			
Happiness	0.05 (-0.04, 0.14)	0.281	0.01 (-0.12, 0.14)	0.875
Depressive symptoms	−0.08 (-0.16, 0.005)	0.065	**0.14(0.04, 0.25**)	**0.006**
Social support	**0.12(0.05, 0.20**)	**0.001**	0.07 (-0.03, 0.17)	0.188

Note. Results weighted with cluster robust standard errors and based on 40 imputed datasets. ATE: average treatment effect. Coef: coefficient from linear model with standardizedstandardised outcome, meaning coefficients are in standard deviationSD units. Bold text indicates pp<0.05.

In contrast, in ELSA, there was no evidence for an association between group participation and subsequent life satisfaction or happiness (table 3). There was an association between group participation and depressive symptoms, whereby participating in arts and cultural groups was associated with a 0.14 SD higher depressive symptom score compared with not participating (ATE coef=0.14, 95% CI=0.04, 0.25).

### Social support

In JAGES, group participation was associated with better subsequent social support. Participating in groups, compared with not, was associated with a 0.12 SD higher social support score (ATE coef=0.12, 95% CI=0.05, 0.20). In ELSA, there was no evidence for this association.

### Sensitivity analyses

#### Unmeasured confounding

In sensitivity analyses, we computed E-values as indicators of how robust findings were to potential unmeasured confounding (online supplemental table 3). E-values showed that, for significant associations, any unmeasured confounders that were associated with both the exposure and outcome by risk ratios of between 1.16 and 1.54-fold, conditional on the measured covariates, could shift the observed associations to the null. These moderately sized E-values suggest that any unmeasured confounders must be relatively strongly associated with both arts and cultural group participation and well-being outcomes to fully explain the associations, particularly given the wide range of covariates already included.

#### Moderation by sex

Next, we explored whether the associations between arts and cultural group participation and well-being differed according to sex (online supplemental table 4). In JAGES, the associations between group participation and well-being were only present in females. There was evidence that group participation was associated with higher odds of life satisfaction than not participating (ATE OR=1.04, 95% CI=1.004, 1.08). Similarly, also in females, group participation was associated with a 0.16 SD higher social support score (ATE coef=0.16, 95% CI=0.07, 0.25) and a 0.10 SD lower depressive symptom score (ATE coef=−0.10, 95% CI=-0.19, 0.00]. There was no evidence for these associations in males.

In ELSA, the associations also differed between males and females. In contrast to JAGES, arts and cultural group participation was associated with lower odds of life satisfaction in males (ATE OR=0.94, 95% CI=0.89, 1.00). This OR translated to 91.5% of males in the treatment group being satisfied with life, relative to 91.9% of males in the control group at baseline. There was no evidence for an association with life satisfaction in females. Instead, in females, group participation was associated with both higher happiness ratings (ATE coef=0.16, 95% CI=0.02, 0.29) and higher depressive symptoms (ATE coef=0.16, 95% CI=0.02, 0.31) compared with not participating.

#### ELSA participants aged 50 years and over

Finally, we replicated the main analyses in the complete ELSA sample, including all participants aged 50 years and over (n=5128; online supplemental table 5). As this larger sample included younger participants, it was no longer comparable with the JAGES sample (online supplemental table 6). There was no evidence for an association between group participation and depressive symptoms. Instead, there was only evidence that participating in an arts and cultural group was associated with a 0.10 SD higher social support score (ATE coef=0.10, 95% CI=0.01, 0.19).

Further exploratory sensitivity analyses, available on request, confirmed that results were not altered by (a) removing self-rated health as a covariate, as it may be on the causal pathway; (b) additionally adjusting for social contact with family members, which is unlikely to be on the causal pathway, but could influence group participation; (c) applying a square root transformation to depressive symptoms, as they were positively skewed; (d) using a binary indicator of depression diagnoses or negative affect, both common approaches to modelling CES-D scores;^21^ (e) limiting the samples to those without depressive symptoms at baseline, as the associations may not have been present in healthy participants; and (f) analysing life satisfaction continuously in ELSA.

## Discussion

In this study, we performed the first cross-country comparison of the effects of arts and cultural groups on well-being. In Japan and England, between 14% and 16% of older adults participated in an arts and cultural group. In Japan, this participation was associated with small increases in the odds of life satisfaction and more social support 3 years later, with the associations strongest in women. In England, group participation was associated with a small increase in social support 4 years later, but only when including all participants aged 50 years and over. The relationship was not present in adults aged 65 years and over. Instead, group participation in adults aged 65 years and over was associated with higher depressive symptoms 4 years later, specifically in females. These results were not maintained when the sample was extended to include those aged 50 years and over.

The evidence for a small effect of arts and cultural group participation on social support was consistent across Japan and the English sample aged 50 years and over. Given that we accounted for previous levels of social support in analyses, this indicates that participating in groups can help to prevent a decline in social support. There are various mechanisms through which this may occur. Arts and cultural groups are likely to provide older adults with opportunities for increasing social contact, meeting like-minded people, enhancing social engagement and building social capital.^7^ Groups can provide older adults with a social identity, the knowledge that they belong to a certain group together with an emotional value or significance attached to that group.^51^ Those who share a social identity are more likely to give and receive social support, thus increasing the perceived social support of group members.^52^ In Japan, these associations were only present in females, which may be because they are more likely to participate in arts and cultural groups.^28^ This may also reflect gender differences in social support and social networks as people age. Men tend to maintain close relationships with fewer people as they age compared with women, with differential effects on health outcomes.^53^,^55^

In Japan, there was also evidence that arts and cultural group participation led to higher odds of life satisfaction, alongside a weak association with lower depressive symptom levels. Although the association with life satisfaction was small, it is consistent with previous population-level evidence from the USA, UK and Italy.^19 21 22^ In contrast, we found no evidence for an association with life satisfaction in England. This could have been due to the relative stability of life satisfaction over time in England. In Japan, the proportion of older adults satisfied with life decreased from 87% at baseline to 83% at follow-up, whereas in England, 91% were satisfied across waves. In sensitivity analyses including English males aged 50 years and over, group participation was actually associated with lower odds of being satisfied with life at follow-up. Yet, given the reduced sample size in this sensitivity analysis, it is possible that this was a false positive.

Additionally, although group participation was weakly associated with fewer depressive symptoms in Japan, it was associated with more depressive symptoms in those aged 65 years and over in England. There are several potential explanations for this finding. Previous research has found that engagement in arts and cultural activities can reduce depressive symptoms in older adults in Japan^13 15^ and England,^56^,^58^ even after accounting for the role of socioeconomic status. Yet, participation must be sustained over time for the largest effects on well-being.^14 59^ As we only measured group participation at one timepoint, we could not assess whether this was short- or long-term engagement, which may have masked any benefits. Similarly, the frequency of group participation may have influenced our findings, but we were not able to measure it. For example, it is possible that participation in Japan was more frequent than in England, explaining the differences in results. It is additionally possible that older adults in England who participate in arts and cultural groups have smaller social networks, going to these groups instead of spending time with family members, which might increase the risk of depression. We tested this explanation in sensitivity analyses, but the results were not altered after adjusting for familial social contact.

However, after including all English participants aged 50 years and over, the association with depressive symptoms was no longer present. Looking at the characteristics of these participants, they were healthier, more highly educated and less likely to be retired than those aged 65 years and over on average (online supplemental table 6). Given that these factors were all included as covariates, we would not expect differences to affect the associations between group participation and well-being. Yet, it is possible that they are moderators. For example, healthier people might be more able to fully participate in arts and cultural groups and thus experience more benefits. There were also other differences between these samples (on factors that we did not include as covariates), such as older participants being more likely to be troubled with pain and to report poor memory. This could mean that attending arts and cultural groups is a difficult and thus negative experience for them. Additionally, exploring gender differences in all English participants aged 50 years and over, the negative association with depressive symptoms was only present in women, for whom there was also an association between group participation and higher happiness. The findings therefore are not consistent across domains and require further investigation with more detailed measures of subjective well-being.

Although mixed, our findings indicate that participating in arts and cultural groups may enhance older adults’ social support across Japan and England. This is promising as it indicates that such participation could have beneficial impacts on populations in different countries and cultures. Increasing well-being may also have subsequent positive impacts on physical and mental health, by reducing stress, depressive symptoms and chronic disease as well as enhancing optimism, self-efficacy, immune function and engagement in other health behaviours.^4 60^ Understanding the role of arts and cultural groups in well-being is particularly important given the increasing interest in social prescribing, which is being implemented in the UK as an accessible and cost-effective strategy to support health.^61^ In Japan, despite increasing interest in social prescribing, there is not yet sufficient evidence or support for its implementation nationally.^62 63^ We present the first evidence that arts and cultural activities may enhance well-being in Japan, although effect sizes were small. Further research should explore other health outcomes to provide further support for the implementation of social prescribing schemes, which could alleviate inequalities in engagement and ensure that all older adults have opportunities for participation.

This study has several strengths, including being the first to compare the associations between arts and cultural group participation and well-being across countries. Using two national population-based studies, we had rich data on demographic, socioeconomic, neighbourhood and health-related factors. ELSA includes a large sample of older adults from England, weighted to be nationally representative, and applying population weights enhanced the representativeness of the JAGES sample. We included multiple measures of subjective well-being and social support, standardising outcomes to allow for comparison across domains. However, this study also has limitations. Sample sizes varied substantially across countries (9511 in Japan, 3133 in England), which could have meant our study was underpowered to detect associations in England. We used IPWRA, a doubly robust approach that provides unbiased estimates when either the treatment or outcome model is misspecified. Our sensitivity analyses indicated that any unmeasured confounders would need to be relatively strongly associated with group participation and well-being to fully explain the associations.^41^ Although we did include a wide range of confounders, unmeasured confounding of this strength remains plausible, which could mean that neither our treatment nor outcome model was correctly specified. In this case, our findings could be a result of residual and unmeasured confounding, as it is unclear which estimator is most appropriate.^64^ Nevertheless, IPWRA provided a more robust approach than traditional regression models. Additionally, happiness and life satisfaction were measured with single items. Although recommended by the UK’s Office for National Statistics,^36^ it is possible that these measures were not sensitive enough to capture effects of group participation 3 to 4 years earlier. This is particularly likely for life satisfaction, which was measured with a binary (yes/no) question in JAGES. Analyses should be repeated with more in-depth measures of life satisfaction and happiness. Depressive symptoms were measured with different scales across countries (GDS in Japan vs CES-D in England). Both are validated screening instruments, but this could have explained the contrasting findings across countries, as the GDS may be better at detecting lower levels of depressive symptoms.^65^ Finally, further research should explore the associations between group participation and social support using more detailed measures, including how often participants feel that they can open up to others as well as other aspects of social support.

## Conclusion

In this study, we estimated the effect of participating in arts and cultural groups on subsequent subjective well-being and social support in Japan and England. Our findings indicate that arts and cultural group participation can enhance life satisfaction and social support in Japan, perhaps with more consistent benefits than in England. The mixed evidence for the associations between group participation and subjective well-being indicates the need for further research with more detailed multidimensional measures of well-being. Across countries, arts and cultural group participation was most clearly associated with social support. Therefore, despite differences in artistic expression, aesthetic experiences, arts administration and funding, arts and cultural group participation may enable older adults to maintain a healthy social support network across countries, which could further support their health as they age.

## supplementary material

10.1136/bmjph-2023-000865 online supplemental file 1

## Data Availability

Data may be obtained from a third party and are not publicly available.

## References

[1] Cabinet Office Japan (2021). Annual Report on the Ageing Society Summary FY2021.

[2] World Health Organization (2020). Decade of healthy ageing: baseline report.

[3] Ryan RM, Deci EL (2001). On happiness and human potentials: A review of research on hedonic and eudaimonic well-being. Annu Rev Psychol.

[4] Keyes CLM (1998). Social Well-Being. Soc Psychol Q.

[5] World Health Organization (2015). World report on ageing and health.

[6] Dunphy K, Baker FA, Dumaresq E (2018). Creative Arts Interventions to Address Depression in Older Adults: A Systematic Review of Outcomes, Processes, and Mechanisms. Front Psychol.

[7] Fancourt D, Aughterson H, Finn S (2021). How leisure activities affect health: a narrative review and multi-level theoretical framework of mechanisms of action. Lancet Psychiatry.

[8] Noice T, Noice H, Kramer AF (2014). Participatory arts for older adults: A review of benefits and challenges. Gerontol.

[9] Yuko Suda (2017). Changes in Citizen Participation in Japanese Civil Society. JMC.

[10] Nakagawa K, Kawachi I (2019). What types of activities increase participation in community “salons”?. Soc Sci Med.

[11] Watanabe R, Kondo K, Saito T (2019). Change in Municipality-Level Health-Related Social Capital and Depressive Symptoms: Ecological and 5-Year Repeated Cross-Sectional Study from the JAGES. Int J Environ Res Public Health.

[12] Saito J, Haseda M, Amemiya A (2019). Community-based care for healthy ageing: lessons from Japan. Bull World Health Organ.

[13] Noguchi T, Ishihara M, Murata C (2022). Art and cultural activity engagement and depressive symptom onset among older adults: A longitudinal study from the Japanese Gerontological Evaluation Study. Int J Geriatr Psychiatry.

[14] Shiba K, Torres JM, Daoud A (2021). Estimating the Impact of Sustained Social Participation on Depressive Symptoms in Older Adults. Epidemiology.

[15] Yamaguchi M, Inoue Y, Shinozaki T (2019). Community Social Capital and Depressive Symptoms Among Older People in Japan: A Multilevel Longitudinal Study. J Epidemiol.

[16] Bone JK, Fancourt D (2022). Arts Culture & the Brain.

[17] Fancourt D, Finn S (2019). What is the evidence on the role of the arts in improving health and well-being? A scoping review. https://europepmc.org/article/NBK/nbk553773.

[18] Daykin N, Mansfield L, Meads C (2018). What works for wellbeing? A systematic review of wellbeing outcomes for music and singing in adults. Perspect Public Health.

[19] Bone JK, Fancourt D, Fluharty ME (2023). Associations between participation in community arts groups and aspects ofwellbeing in older adults in the United States: A propensity score matching analysis. *Aging Ment Health*.

[20] Menec VH (2003). The relation between everyday activities and successful aging: A 6-year longitudinal study. J Gerontol B Psychol Sci Soc Sci.

[21] Fancourt D, Steptoe A (2018). Community group membership and multidimensional subjective well-being in older age. J Epidemiol Community Health.

[22] Grossi E, Tavano Blessi G, Sacco PL (2012). The Interaction Between Culture, Health and Psychological Well-Being: Data Mining from the Italian Culture and Well-Being Project. J Happiness Stud.

[23] Cutler D (2015). Living national treasure - arts and older people in Japan.

[24] Dow R, Warran K, Letrondo P (2023). The arts in public health policy: progress and opportunities. Lancet Public Health.

[25] Naoe T (2003). Art Education in Lower Secondary Schools in Japan and the United Kingdom. J Aesth Educ.

[26] Bone JK, Bu F, Fluharty ME (2021). Who engages in the arts in the United States? A comparison of several types of engagement using data from The General Social Survey. BMC Public Health.

[27] Mak HW, Coulter R, Fancourt D (2020). Patterns of social inequality in arts and cultural participation: Findings from a nationally representative sample of adults living in the United Kingdom of Great Britain and Northern Ireland. Public Health Panor.

[28] Bone JK, Noguchi T, Mak HW (2023). Predictors of arts engagement in older adults in japan: novel findings from a national study and cross-cultural comparisons with the united states. PsyArXiv.

[29] Ryff CD (2017). Eudaimonic well-being, inequality, and health: Recent findings and future directions. *Int Rev Econ*.

[30] Shah BR, Laupacis A, Hux JE (2005). Propensity score methods gave similar results to traditional regression modeling in observational studies: A systematic review. J Clin Epidemiol.

[31] Wang S, Mak HW, Fancourt D (2020). Arts, mental distress, mental health functioning & life satisfaction: fixed-effects analyses of a nationally-representative panel study. BMC Public Health.

[32] Węziak-Białowolska D (2016). Attendance of cultural events and involvement with the arts-impact evaluation on health and well-being from a Swiss household panel survey. Public Health (Fairfax).

[33] Ho AHY, Ma SHX, Ho M-HR (2019). Arts for ageing well: A propensity score matching analysis of the effects of arts engagements on holistic well-being among older Asian adults above 50 years of age. BMJ Open.

[34] Steptoe A, Fancourt D (2019). Leading a meaningful life at older ages and its relationship with social engagement, prosperity, health, biology, and time use. Proc Natl Acad Sci U S A.

[35] Kondo K (2016). Progress in Aging Epidemiology in Japan: The JAGES Project. J Epidemiol.

[36] Office for National Statistics (2022). Personal well-being in the UK: april 2021 to march 2022.

[37] Vanhoutte B (2014). The Multidimensional Structure of Subjective Well-Being In Later Life. J Popul Ageing.

[38] Office for National Statistics (2011). Initial investigation into Subjective Well-being from the Opinions Survey.

[39] Yesavage JA, Sheikh JI (1986). 9/Geriatric Depression Scale (GDS): Recent evidence and development of a shorter version. Clin Gerontol.

[40] Turvey CL, Wallace RB, Herzog R (1999). A revised CES-D measure of depressive symptoms and A DSM-based measure of major depressive episodes in the elderly. Int Psychogeriatr.

[41] Funk MJ, Westreich D, Wiesen C (2011). Doubly Robust Estimation of Causal Effects. Am J Epidemiol.

[42] StataCorp (2021). Stata statistical software: release 17.

[43] Sauzet O, Wright KC, Marston L (2013). Modelling the hierarchical structure in datasets with very small clusters: A simulation study to explore the effect of the proportion of clusters when the outcome is continuous. Stat Med.

[44] Japanese Government Statistics (2020). Population Census 2020.

[45] Hainmueller J, Xu Y (2013). Ebalance: A stata package for entropy balancing. J Stat Softw.

[46] Chowdhury S, Khare M, Wolter K (2007). Weight trimming in the national immunization survey.

[47] Potter F, Zheng Y (2015). Methods and issues in trimming extreme weights in sample surveys.

[48] White IR, Royston P, Wood AM (2011). Multiple imputation using chained equations: Issues and guidance for practice. Stat Med.

[49] VanderWeele TJ, Ding P (2017). Sensitivity Analysis in Observational Research: Introducing the E-Value. Ann Intern Med.

[50] Linden A, Mathur MB, VanderWeele TJ (2020). Conducting sensitivity analysis for unmeasured confounding in observational studies using E-values: The evalue package. Stata J.

[51] Hogg MA, Burke PJ (2006). Contemporary Social Psychological Theories.

[52] Haslam SA, Jetten J, Postmes T (2009). Social Identity, Health and Well‐Being: An Emerging Agenda for Applied Psychology. Appl Psychol.

[53] Antonucci TC, Akiyama H (1987). An examination of sex differences in social support among older men and women. Sex Roles.

[54] Shye D, Mullooly JP, Freeborn DK (1995). Gender differences in the relationship between social network support and mortality: A longitudinal study of an elderly cohort. Soc Sci Med.

[55] Vaux A (1985). Variations in Social Support Associated with Gender, Ethnicity, and Age. J Soc Issues.

[56] Fancourt D, Tymoszuk U (2019). Cultural engagement and incident depression in older adults: evidence from the English Longitudinal Study of Ageing. Br J Psychiatry.

[57] Fancourt D, Opher S, de Oliveira C (2020). Fixed-Effects Analyses of Time-Varying Associations between Hobbies and Depression in a Longitudinal Cohort Study: Support for Social Prescribing?. Psychother Psychosom.

[58] Fancourt D, Steptoe A (2019). Cultural engagement and mental health: Does socio-economic status explain the association?. Soc Sci Med.

[59] Tymoszuk U, Perkins R, Spiro N (2020). Longitudinal Associations Between Short-Term, Repeated, and Sustained Arts Engagement and Well-Being Outcomes in Older Adults. The J Gerontol.

[60] Berkman LF, Glass T, Brissette I (2000). From social integration to health: Durkheim in the new millennium. Soc Sci Med.

[61] Drinkwater C, Wildman J, Moffatt S (2019). Social prescribing. BMJ.

[62] Nishioka D, Kondo N (2020). A Literature Review of Social Prescribing: Implementation Challenges and Opportunities in Japan. Iryo Shakai.

[63] Cabinet Office (2021). Basic Policy on Economic and Fiscal Management and Reform 2021.

[64] Tsiatis AA, Davidian M (2007). Comment: Demystifying Double Robustness: A Comparison of Alternative Strategies for Estimating A Population Mean from Incomplete Data. Stat Sci.

[65] Lyness JM, Noel TK, Cox C (1997). Screening for depression in elderly primary care patients. A comparison of the Center for Epidemiologic Studies-Depression Scale and the Geriatric Depression Scale. Arch Intern Med.

