# Supplementary material for: Does arts and cultural group participation influence subsequent well-being? A longitudinal cross-country comparison of older adults in Japan and England
Source: BMJ Public Health. 2024 Aug 24;2(2):e000865. doi: 10.1136/bmjph-2023-000865 (PMC11816965; doi:10.1136/bmjph-2023-000865)
Supplement: online supplemental file 1 [file bmjph-2-2-s001.pdf]

Supplementary Materials

Supplementary methods

Table S1. Measurement and harmonization of all variables across datasets.

Variable	Japan (JAGES)	England (ELSA)
**Exposure (measured at baseline)**		
Community arts and cultural group participation	Binary indicator (0,1). Maximum score on:<br>1. *How often do you attend activities of learning or cultural groups?*<br>*0 = never, a few times a year*<br>*1 = one to three times a month, once a week, two or three times a week, four or more times a week*<br>2. *For participants who reported currently participating in community activities held by the local government or social welfare council. Please indicate not more than three activities or programs you have most frequently participated in:*<br>*0 = calisthenics, health-consciousness lecture, interaction with younger people, teatime and chitchat, brain exercises, indoor games, other*<br>*1 = crafting (handicrafts), musical activities (singing, playing musical instruments, etc.), hobbies (calligraphy, haiku, senryu [traditional Japanese poetry], etc.)*	Binary indicator (0,1)<br>*Are you a member of education, arts or music groups or evening classes?*<br>*0 = No*<br>*1 = Yes*
**Outcomes (measured at baseline and follow-up)**		
Social wellbeing	Index of social support, higher scores indicate more social support (0-5). Total of:<br>1. *How often do you see your friends?*<br>*0 = less than monthly (hardly/never, a few times a yar)*<br>*1 = at least monthly (1-3 times a month, 1 times a week, 2-3 times a week, 4+ times a week)*<br>*Do you have someone who listens to your concerns and complaints? (1 point for each of the following circled)*<br>2. *Spouse*<br>3. *Children living together, children or relatives living apart*<br>4. *Brother, sister, relative, parents, grandchildren*<br>5. *Friend*	Index of social support, higher scores indicate more social support (0-5). Total of:<br>1. *How often do you meet up with your friends?*<br>*0 = less than monthly (< once a year or never, once or twice a year, every few months)*<br>*1 = at least monthly (once or twice a month, once or twice a week, three or more times a week)*<br>*How much can you open up to the following people if you need to talk? (For each: 0 = not at all, 1 = a little, some, a lot)*<br>2. *Spouse*<br>3. *Children*<br>4. *Other relatives*<br>5. *Friends*
Subjective wellbeing: Happiness	*To what degree do you feel you are currently happy?*<br>*Rated very unhappy (0) to very happy (10)*<br>Standardised score	Overall, how happy did you feel yesterday?<br>*Rated not at all (0) to very (10)*<br>Standardised score

Variable	Japan (JAGES)	England (ELSA)
Subjective wellbeing:<br>Life satisfaction	*Are you satisfied with your current life?*<br>Satisfied (yes)<br>Not satisfied (no)	*Overall, how satisfied are you with your life nowadays?*<br>*Rated not at all (0) to very (10)*<br>Satisfied (rating of 5-10)<br>Not satisfied (rating of 0-4)
Subjective wellbeing:<br>Depressive symptoms	*Geriatric Depression Scale (15-items), higher scores indicate more depressive symptoms (0-15)*<br>Standardised score	*Center for Epidemiologic Studies Depression Scale (8 items), higher scores indicate more depressive symptoms (0-8)*<br>Standardised score
**Covariates (measured at baseline)**		
Age	Continuous years	Continuous years
Sex	Male<br>Female	Male<br>Female
Ethnicity	-	White<br>Other
Marital status	Married<br>Widowed<br>Other (or divorced, never married)	Married (or civil partnership, remarried)<br>Widowed<br>Other (or divorced, separated, never married)
Household size	Continuous number of people in the household	Continuous number of people living in household
Years of education	≤9 years<br>10-12 years<br>≥13 years	≤9 years<br>10-12 years<br>≥13 years
Employment status	Employed<br>Unemployed (never had a job, looking for a job)<br>Retired	Employed (or self-employed)<br>Unemployed (or permanently sick or disabled, looking after home or family)<br>Retired
Total assets	*What is the total value of your household assets, including savings, real estate (e.g. house, land, condominium), stocks, golf membership, etc.?*<br><¥5 million<br>¥5-¥9.99 million<br>¥10-¥49.99 million<br>≥¥50 million	*ELSA total assets calculated variable including savings, investments, real estate, business assets, artwork, jewellery, debt, etc.*<br><£99,999<br>£100,000-£249,999<br>£250,000-£499,999<br>£500,000+
Home ownership	Home owner (own detached house, own apartment or condominium)<br>Non home owner (rent apartment from a public body, rent non-governmental detached house, rent non-governmental apartment or condominium, rental house, other)	Home owner (own outright, buying with mortgage or loan, shared ownership)<br>Non home owner (rent, live rent free, squatting)
Subjective financial status	*Current financial situation in light of general economic conditions:*<br>Difficult (or very difficult)<br>Average<br>Comfortable (or very comfortable)	*How respondent (and partner) getting along financially these days:*<br>Difficult (don't manage very well, have some financial difficulties, or have severe financial difficulties)<br>Average (get by alright)<br>Comfortable (manage very well, manage quite well)

Variable	Japan (JAGES)	England (ELSA)
Equivalised household income	Continuous ¥100,000 units divided by √n household members<br><br>*Midpoint of each category taken as continuous indicator from: What was your pretax annual household net income for 2015 (including pension)?*<br>*1. Less than 500,000 yen*<br>*2. 500,000 to less than 1 million yen*<br>*3. 1 million to less than 1.5 million yen*<br>*4. 1.5 million to less than 2 million yen*<br>*5. 2 million to less than 2.5 million yen*<br>*6. 2.5 million to less than 3 million yen*<br>*7. 3 million to less than 4 million yen*<br>*8. 4 million to less than 5 million yen*<br>*9. 5 million to less than 6 million yen*<br>*10. 6 million to less than 7 million yen*<br>*11. 7 million to less than 8 million yen*<br>*12. 8 million to less than 9 million yen*<br>*13. 9 million to less than 10 million yen*<br>*14. 10 million to less than 12 million yen*<br>*15. More than 12 million yen*	Continuous £1000 units divided by √n household members<br><br>*ELSA calculated total income including any overtime, bonuses, commissions, tips or tax refund, but before any deductions for tax, national insurance or pension contributions, union dues and so on*
Childhood SES	*What do you think of your living condition at the age of 15 in light of social average around you?*<br>Lower SES (lower, lower middle)<br>Higher SES (upper, upper middle, middle)	*Whether respondent experienced severe financial hardship in childhood:*<br>Lower SES (yes)<br>Higher SES (no)
Neighbourhood social cohesion	Standardised validated 3-item index, higher scores indicate more cohesion (1-5). Average of:<br>*The following questions are about the area where you live.*<br>*1. Do you think people living in your area can be trusted in general?*<br>*Very / Moderately / Neutral / Not much / Not at all*<br>*2. Do you think people living in your area try to help others in the most of situations?*<br>*Very / Moderately / Neutral / Not much / Not at all*<br>*3. How attached are you to the area you live?*<br>*Very / Moderately / Neutral / Not much / Not at all*	Standardised validated 4-item index, higher scores indicate more cohesion (1-7). Average of:<br>*How do you feel about your local area, that is everywhere within a 20 minute walk or about a mile of your home? Please tick one box on each line. The closer your tick is to a statement the more strongly you agree with it.*<br>*1. I really feel part of this area (1) - I feel that I don't belong in this area (7)**<br>*2. Most people in this area can be trusted (1) - Most people in this area can't be trusted (7)**<br>*3. Most people in this area are friendly (1) - Most people in this area are unfriendly (7)**<br><br>*4. If you were in trouble, there are lots of people in this area who would help you (1) - If you were in trouble, there is nobody in this area who would help you (7)**<br>**reverse scored*

Variable	Japan (JAGES)	England (ELSA)
Neighbourhood area physical disorder	Standardised 2-item physical disorder index, higher scores indicate more disorder (1-4). Measured in wave before baseline. Average of:<br>*Are the following present within walking distance of your home (within about 1 km)?*<br>1. *Locations with noticeable graffiti or undisposed garbage*<br>*Many / Some / Few / None*<br>2. *Dangerous places when walking alone at night*<br>*Many / Some / Few / None*	Standardised 3-item physical disorder index, higher scores indicate more disorder (1-7). Average of:<br>*How do you feel about your local area, that is everywhere within a 20 minute walk or about a mile of your home? Please tick one box on each line. The closer your tick is to a statement the more strongly you agree with it.*<br>1. *Vandalism and graffiti are a big problem in this area (1) - There is no problem with vandalism and graffiti in this area (7)**<br>2. *People would be afraid to walk alone after dark in this area (1) - People feel safe walking alone in this area after dark (7)**<br>3. *This area is kept very clean (1) - This area is always full of litter and rubbish (7)*<br>**reverse scored*
General health rating	*Self-reported general health*<br>Excellent<br>Good<br>Fair or poor	*Self-reported general health*<br>Excellent<br>Good (good, very good)<br>Fair or poor
Long-term health conditions	Continuous number of conditions (0-8)<br>*From: high blood pressure, diabetes, cancer, respiratory disease, heart disease, stroke, dementia/Alzheimer's, Parkinson's disease*	Continuous number of conditions (0-8)<br>*From: high blood pressure, diabetes, cancer, lung disease, heart disease, stroke, dementia/Alzheimer's, Parkinson's disease*
Psychiatric problems	-	*Have you ever been told by a told by a doctor that you have emotional, nervous or psychiatric problems?*<br>No<br>Yes
Depression diagnosis	*Circle the number of all diseases for which you are currently receiving treatment or experiencing after-effects.*<br>Depression	*What type of emotional, nervous or psychiatric problems do you have?*<br>Depression
Difficulties with activities of daily living (ADLs)	Continuous number of activities with which participant has difficulties (0-2)<br>*From: walking about, washing or dressing*	Continuous number of activities with which participant has difficulties (0-2)<br>*From: walking 100 yards, washing or dressing*
Difficulties with instrumental activities of daily living (IADLs)	Continuous number of activities with which participant has difficulties (0-4)<br>*From: cook for self, shopping for daily necessities, make calls yourself, pay bills yourself*	Continuous number of activities with which participant has difficulties (0-4)<br>*From: prepare hot meals, shopping for groceries, making telephone calls, managing money (e.g. paying bills and keeping track of expenses)*

Note: Items from JAGES are shown translated into English. Original Japanese questions are available from the corresponding author on reasonable request.

Probability weights

We applied weights to make the samples representative of Japanese and English older adults. Probability weights were not available for JAGES. We therefore weighted the final analytical sample to match the characteristics of the Japanese population aged 65 and over according to age, sex, marital status, and number of household members (obtained from the population census; Japanese Government Statistics, 2020) using the Stata package *ebalance* (Hainmueller & Xu, 2013). To remove extreme variation, weights were trimmed to a maximum of the median plus six times the interquartile range, and then adjusted so that the total summed to the number of participants (Chowdhury et al., 2007; Potter & Zheng, 2015). In ELSA, we used probability weights for the self-completion questionnaire provided in the data, which account for complex sampling strategies and non-response.

Additional exploratory sensitivity analyses

Further exploratory sensitivity analyses, available on request, confirmed that results were not altered by: a) removing self-rated health as a covariate, as it may be on the causal pathway; b) additionally adjusting for social contact with family members, which is unlikely to be on the causal pathway, but could influence group participation; c) applying a square root transformation to depressive symptoms, as they were positively skewed; d) using a binary indicator of depression diagnoses or negative affect, both common approaches to modelling CES-D scores (Fancourt & Steptoe, 2018); e) limiting the samples to those with no depressive symptoms at baseline, as the associations may not have been present in healthy participants; and f) analyzing life satisfaction continuously in ELSA.

Table S2. Number of participants with missing data for each variable of interest.

	**JAGES** n=9,511	**ELSA** n=3,133
Education	102 (1%)	4 (<1%)
Employment status	1405 (15%)	-
Household income	1068 (11%)	38 (1%)
Total assets	1684 (18%)	38 (1%)
Home owner	69 (1%)	3 (<1%)
Subjective financial status	40 (<1%)	149 (5%)
Higher childhood SES	558 (6%)	1218 (39%)
Neighborhood social cohesion	162 (2%)	61 (2%)
Neighborhood physical disorder	2406 (25%)	65 (2%)
General health	211 (2%)	1 (<1%)
Long-term health conditions	440 (5%)	-
Self-reported depression diagnosis	440 (5%)	18 (<1%)
Difficulties with ADLs	480 (5%)	-
Difficulties with IADLs	218 (2%)	-
Arts and cultural group participation	1658 (17%)	155 (5%)
Baseline life satisfaction	224 (2%)	117 (4%)
Baseline happiness	202 (2%)	163 (5%)
Baseline depressive symptoms	1421 (15%)	7 (<1%)
Baseline social support	232 (2%)	314 (10%)
Follow-up life satisfaction	293 (3%)	66 (2%)
Follow-up happiness	378 (4%)	185 (6%)
Follow-up depressive symptoms	1567 (16%)	6 (<1%)
Follow-up social support	324 (3%)	380 (12%)

Note. There were no missing data in age, sex, marital status, or household size across cohorts, or in psychiatric diagnosis in ELSA.

Supplementary results

Table S3. E-values indicating how robust the main findings were to potential unmeasured confounding.

	**JAGES**		**ELSA**	
	ATE E-value	Confidence interval	ATE E-value	Confidence interval
Life satisfaction	**1.16**	**1.04**	1.09	1.00
Happiness	1.27	1.00	1.11	1.00
Depressive symptoms	1.35	1.00	**1.54**	**1.24**
Social support	**1.49**	**1.28**	1.32	1.00

Note. Bold text indicates associations where $p<0.05$ in the main analyses. Confidence intervals closest to the null are shown.

Table S4. Results from inverse probability weighted regression adjustment estimating the effect of group arts engagement on subsequent wellbeing estimated separately by sex.

	**JAGES**				**ELSA**			
	**Males** (n=4,485)		**Females** (n=5,026)		**Males** (n=2,238)		**Females** (n=2,890)	
	ATE [95% CI]	p value	ATE [95% CI]	p value	ATE [95% CI]	p value	ATE [95% CI]	p value
	Binary outcome: Odds Ratio							
Life satisfaction	1.03 [0.98, 1.09]	0.254	**1.04 [1.004, 1.08]**	**0.029**	**0.94 [0.89, 1.00]**	**0.046**	1.01 [0.95, 1.07]	0.814
	Continuous outcome: Coef							
Happiness	0.04 [-0.11, 0.18]	0.609	0.09 [-0.02, 0.19]	0.117	0.00 [-0.15, 0.15]	0.985	**0.16 [0.02, 0.29]**	**0.026**
Depressive symptoms	-0.07 [-0.19, 0.05]	0.243	-0.10 [-0.19, 0.00]	0.051	-0.03 [-0.15, 0.10]	0.688	**0.16 [0.02, 0.31]**	**0.028**
Social support	0.07 [-0.05, 0.18]	0.269	**0.16 [0.07, 0.25]**	**0.001**	0.11 [-0.01, 0.23]	0.076	0.09 [-0.02, 0.19]	0.096

Note. Results weighted with cluster robust standard errors and based on 40 imputed datasets. ATE: average treatment effect. Coef: coefficient from linear model with standardized outcome, meaning coefficients are in standard deviation units. ELSA sample includes all participants aged 50 and above. Also note differences in ELSA life satisfaction models for males (childhood SES not included) and females (education binary; ≤12 vs ≥13). Bold text indicates $p<0.05$.

Table S5. Results from inverse probability weighted regression adjustment estimating the effect of group arts engagement on subsequent wellbeing in all ELSA participants aged 50 and above (n=5,128).

	**ATE [95% CI]**	**p value**	**E-value [CI]**
	Odds ratio		
Life satisfaction	0.99 [0.95, 1.03]	0.558	1.09 [1.00]
	Coef		
Happiness	0.08 [-0.03, 0.19]	0.172	1.35 [1.00]
Depressive symptoms	0.08 [-0.02, 0.17]	0.121	1.35 [1.00]
Social support	**0.10 [0.01, 0.19]**	**0.025**	**1.42 [1.12]**

Note. Results weighted with cluster robust standard errors and based on 40 imputed datasets. ATE: average treatment effect. Coef: coefficient from linear model with standardized outcome, meaning coefficients are in standard deviation units. Bold text indicates $p<0.05$.

Table S6. Descriptive statistics after imputation and weighting for both ELSA samples: main analytical sample (aged 65 and above) and full sample in sensitivity analyses (aged 50 and above).

	**Aged 65+** n=3,133	**Aged 50+** n=5,128
	**Mean (SD)**	
Age	72.91 (6.34)	64.40 (10.50)
Household size	1.88 (0.72)	2.15 (0.95)
Household income	£19.88 (£14.14)	£20.99 (£14.91)
Neighborhood social cohesion	5.59 (1.23)	5.45 (1.28)
Neighborhood deprivation	2.64 (1.23)	2.68 (1.23)
Long-term health conditions	1.11 (0.97)	0.84 (0.99)
Difficulties with ADLs	0.22 (0.53)	0.18 (0.55)
Difficulties with IADLs	0.12 (0.43)	0.10 (0.54)
	**Proportion**	
Participated in arts group	14%	12%
Female	54%	52%
Marital status		
Married	68%	68%
Widowed	17%	9%
Other	15%	23%
Education		
≤9 years	10%	6%
10-12 years	60%	54%
≥13 years	30%	40%
Employment status		
Employed	13%	44%
Unemployed	5%	9%
Retired	82%	47%
Total assets		
1	17%	21%
2	29%	28%
3	31%	29%
4	22%	22%
Home owner	84%	83%
Subjective financial status		
Difficult	2%	5%
Average	19%	22%
Comfortable	79%	73%
Higher childhood SES	96%	96%
General health		
Excellent	9%	14%
Good	67%	64%
Fair/poor	24%	22%
Psychiatric diagnosis	9%	11%
Self-reported depression diagnosis	5%	8%
Self-reported long-standing illness	57%	49%
Used day center in the last month	0.3%	0.3%
Often troubled with pain	42%	38%
Self-rated memory		
Excellent/very good	17%	23%
Good	46%	46%
Fair/poor	37%	31%

Note. Results weighted with cluster robust standard errors and based on 40 imputed datasets. Neighborhood cohesion and deprivation both ranged from 1 to 7. Household income is in units of £10,000 in ELSA. Total asset categories correspond to <100, 100-249, 250-499, ≥500 thousand pounds.

References

Chowdhury, S., Khare, M., & Wolter, K. (2007). Weight trimming in the national immunization survey. In *Proceedings of the Joint Statistical Meetings, Section on Survey Research Methods* (pp. 2651–2658). American Statistical Association.

Fancourt, D., & Steptoe, A. (2018). Community group membership and multidimensional subjective well-being in older age. *Journal of Epidemiology and Community Health*, *72*(5), 376–382. https://doi.org/10.1136/jech-2017-210260

Hainmueller, J., & Xu, Y. (2013). Ebalance: A stata package for entropy balancing. *Journal of Statistical Software*, *54*(7), 1–18. https://doi.org/10.18637/jss.v054.i07

Japanese Government Statistics. (2020). *Population Census 2020*. https://www.e-stat.go.jp/en/stat-search/files?page=1&toukei=00200521&tstat=000001136464

Potter, F., & Zheng, Y. (2015). Methods and Issues in Trimming Extreme Weights in Sample Surveys. In *Proceedings of the American Statistical Association, Section on Survey Research Methods* (pp. 2707–2719). American Statistical Association.
